# Depression and the Glutamate/GABA-Glutamine Cycle

**DOI:** 10.2174/1570159X22666240815120244

**Published:** 2024-08-15

**Authors:** Mortimer Mamelak

**Affiliations:** 1 Department of Psychiatry, Baycrest Hospital, University of Toronto, Toronto, Ontario, Canada

**Keywords:** Depression, stress, glutamate, GABA, glutamine, gammahydroxybutyrate, cholinergic neurotransmission, cerebral metabolic rate

## Abstract

Many features of major depressive disorder are mirrored in rodent models of psychological stress. These models have been used to examine the relationship between the activation of the hypothalamic-pituitary axis in response to stress, the development of oxidative stress and neuroinflammation, the dominance of cholinergic neurotransmission and the associated increase in REM sleep pressure. Rodent models have also provided valuable insights into the impairment of glycolysis and brain glucose utilization by the brain under stress, the resulting decrease in brain energy production and the reduction in glutamate/GABA-glutamine cycling. The rapidly acting antidepressants, scopolamine, ketamine and ECT, all raise extracellular glutamate and scopolamine and ketamine have specifically been shown to increase glutamate/GABA-glutamine cycling in men and rodents with corresponding short-term relief of depression. The nightly use of gammahydroxybutyrate (GHB) may achieve more permanent results and may even act prophylactically to prevent the development or recurrence of depression. GHB is a GABAB agonist and restores the normal balance between cholinergic and monoaminergic neurotransmission by inhibiting cholinergic neurotransmission. It relieves REM sleep pressure. GHB’s metabolism generates NADPH, a key antioxidant cofactor. Its metabolism also generates succinate, the tricarboxylic acid cycle intermediate, to provide energy to the cell and to synthesize glutamate. In both animals and man, GHB increases the level of brain glutamate.

## 1 INTRODUCTION

Psychosocial stress, applied to rodents, is frequently used to model the changes that take place in the human brain in response to severe stress [1-3]. Common paradigms include chronic unpredictable mild stress (CUMS) in which a mouse or a rat is deprived of food or water and exposed to intermittent illumination and tilting of the cage over a period of time. Decreased sucrose preference and reduced social exploration are considered signs of depressive-like behavior. In the learned helplessness model, feelings of despair are created by exposing the animal to a stressful event, *i.e*., a foot shock, that it cannot avoid or control. Restraint stress consists of restricting the free movement of a mouse or rat by placing it in a semi-cylindrical acrylic tube. In chronic social defeat stress, a mouse is challenged and defeated by a larger more aggressive mouse.

## 2 THE STRESS RESPONSE

### 2.1 Acute Stress

In response to acute stress, corticotropin-releasing hormone (CRF) production in the hypothalamus is increased and delivered to the anterior pituitary to stimulate the release of adrenocorticotropic hormone (ACTH) [4]. This, in turn, stimulates the adrenal cortex to secrete cortisol in humans or corticosteroids in rodents. CRF is also released from extra-hypothalamic sites such as the dorsal raphe, lateral dorsal tegmentum and amygdala in response to stress and alters mood, enhances cholinergic activity and disinhibits REM sleep [5-9]. In rodents, the release of corticosteroid, acting through undefined non-genomic and possibly genomic mechanisms, rapidly enhances the excitation of pyramidal neurons in the medial prefrontal cortex (mPFC) and hippocampus (HPC) to increase the level of extracellular glutamate [9-12]. Corticosteroid, by activating glucocorticoid receptors, also induces a delayed and sustained potentiation of the synaptic response by increasing the expression of N-methyl-D-aspartic acid receptors (NMDARs) and α-amino-3-hydroxy-5-methyl-4-isoxazolepropionic acid receptors (AMPARs) on the dendritic spines of PFC pyramidal neurons [11, 13].

### 2.2 Chronic Stress

However, in response to chronic stress or prolonged exposure to corticosteroids, fewer NMDARs and AMPARS are delivered to dendritic spines and glutaminergic transmission is reduced [14]. Chronic stress or corticosteroid reduces the apical dendritic length and branching of mPFC (layers II/III and V) and HPC CA3 pyramidal neurons, while increasing dendritic density in the basolateral amygdala [2]. In prefrontal pyramidal neurons, even acute foot shock stress has been shown to induce long-lasting, 2-week, dendritic atrophy [15]. Dendritic atrophy can be prevented by blocking the actions of glutamate on NMDA receptors and it cannot be induced in genetically modified mice lacking these receptors [10, 16]. Repeated stress results in an overall decrease in mean dendritic spine volume and surface area. The reduction in large spines and the increase in small spines suggests a failure of spinal maturation following repeated stress [17]. Dendritic atrophy and the change in spine morphology reduce dendritic energy consumption and impair synaptic transmission, neural activation and neurotransmitter release [18]. The initial increase in neural activation and glutamate release with stress is followed by a reduction in neural excitation and glutamate release as the system becomes exhausted by unrelieved stress [2, 19].

## 3 PREFRONTAL ENERGY METABOLISM

**Fig. (1):**
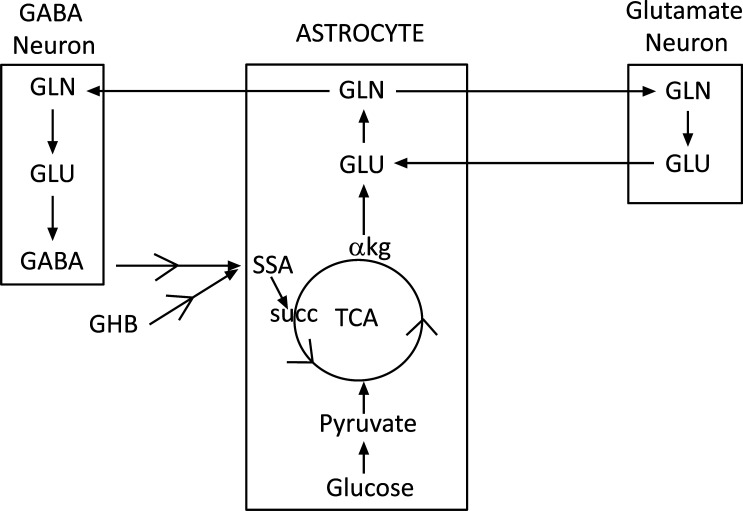
The glutamate/GABA-glutamine cycle. **Abbreviations:** akg: alphaketoglutarate; GABA: gammaaminobutyric acid; GHB gammahydroxybutyratel; GLU: glutamate; GLN glutamine; SUCC: Succinate; ssa: succinicsemialdehyde.

Indeed, studies using CUMS in mice have revealed the profound effects of sustained stress on energy metabolism in the PFC. The cerebral metabolic rate of glucose utilization in this region declines by about 45% together with about a 35% reduction in total ATP production [20]. Energy in the PFC is mostly used for the neurotransmission of glutamate and GABA and specifically for glutamate/GABA/glutamine cycling. The flow of synaptic glutamate and GABA from neurons into astrocytes, and glutamine from astrocytes into neurons describes the glutamine-glutamate/GABA-glutamine cycle [21, 22] (Fig. **1**). An approximate 1:1 ratio between the rates of neuronal glucose oxidation and glutamate/GABA-glutamine neurotransmitter cycling has been established [23, 24]. The flux in this cycle, which is essential for the production of glutamate and GABA, accounts for about 75% of the brain’s glucose utilization [25]. But, with chronic stress, glutamate-glutamine cycling declines by about 47% and GABA-glutamine cycling by about 35% [20]. Metabolic activity is significantly decreased in all 3 major cellular elements of the PFC, the glutamatergic and GABAergic neurons and the astroglia. A very substantial decline in glucose utilization and ATP production occurs in glutamatergic neurons together with a smaller reduction in GABAergic neurons. Glutamate and GABA levels in the PFC are both reduced [20, 26, 27].

### 3.1 Oxidative Stress

What accounts for the reduction in PFC energy metabolism and the impaired production of its two major neurotransmitters? Signs of oxidative stress and neuroinflammation pervade the chronically stressed rodent brain. In rats subject to restraint stress or corticosteroid for 21 days, brain levels of the free radical scavenging antioxidant enzymes superoxide dismutase (SOD), catalase (CAT), glutathione S-transferase (GST) and glutathione reductase (GR) are all reduced. This holds true as well for the key non-enzymatic antioxidant, glutathione (GSH). At the same time, levels of lipid peroxidation and protein carbonyls, two classic markers of oxidative stress, are significantly increased [3]. Mice restrained for 2 hours every day for 14 days develop distinct signs of depressive behavior that persist for 3 months in the absence of intervention. Acute restraint induces a surge of oxidative stress in the brain that increases progressively with the repetition of the stress. Levels of superoxide, lipid peroxidation and protein carbonyls all increase. Restraint stress increases brain corticosteroid levels which, in turn, activates NADPH oxidase and increases the production of reactive oxygen species (vide infra) [3, 28].

### 3.2 Neuroinflammation

Equally striking is the rise in inflammatory mediators in the brain. Specific molecules, known as danger-associated molecular patterns (DAMPs), such as high mobility group box 1 (HMGB1), S100 proteins, histones, heat shock proteins (HSP) and ATP are released in response to oxidative injury and trigger a sterile inflammation [29-31]. Animal models of chronic stress reveal increased microglial activation in the prefrontal and hippocampal regions together with increased cytokine levels such as IL-1β, IL-6, and TNF-α among others [31, 32]. Activation of the NF-κB pathway is a common signaling event that is responsible for the transcriptional induction of pro-inflammatory cytokines and pro-oxidant genes such as the NADPH oxidase NOX2 subunit gp91phox [31, 33]. NADPH oxidase is a major generator of superoxide. Other components of the NADPH oxidase complex, p47phox and p67phox, also increase following stress or the application of corticosteroids and lead to a burst of superoxide production [28]. A self-sustaining cycle of oxidative stress and inflammation is established.

## 4 THE GLUTAMATE/GABA-GLUTAMINE CYCLE

### 4.1 Glycolysis

Oxidative stress and its oxidative products impair cerebral glucose utilization and its capacity to generate energy, ATP, and synthesize its two major neurotransmitters, glutamate and GABA. Glucose utilization is compromised by the oxidative modification of the enzymes of glycolysis, the citric acid cycle and oxidative phosphorylation [34]. Glyceraldehyde-3- phosphate dehydrogenase, a key glycolytic enzyme, is particularly sensitive to oxidative stress and its dysfunction limits the subsequent course of glycolysis, and the production of NADH, ATP and pyruvate [35]. Oxidative stress damages DNA and leads to the activation of Poly (ADP-ribose) polymerase-1 (PARP-1), a sensor of oxidative DNA damage that further limits glycolysis by using and depleting NAD to repair DNA [36]. The glutamate/GABA/-glutamine cycle is critically dependent on glycolysis and the production of NADH and pyruvate [25]. Deficient glycolysis and the insufficient production of NADH may also contribute to the reduced levels of ATP found in the stressed brain [37].

### 4.2 Anaplerosis

In the glutamate/GABA-glutamine cycle, glutamine is synthesized almost exclusively in astrocytes from glutamate formed by the transamination of α-ketoglutarate, again, almost exclusively in astrocytes [21, 22]. Excessive demand for glutamine and glutamate, for example, during intense periods of cerebral activation, drains the TCA cycle of α-ketoglutarate and disrupts oxidative phosphorylation and energy production. To prevent this depletion, pyruvate carboxylase, the primary anaplerotic enzyme of the brain, found only in astrocytes, converts one of the two molecules of pyruvate generated by glycolysis to oxaloacetate. Pyruvate dehydrogenase converts the other pyruvate molecule to acetyl-CoA which then combines with oxaloacetate to form the TCA intermediate citrate. Citrate is then metabolized along the TCA to replenish α-ketoglutarate. Although pyruvate carboxylation is necessary to restore α-ketoglutarate levels, its real function is to generate glutamate.

### 4.3 Malate Aspartate Shuttle

The two molecules of NADH formed in the cytoplasm by glycolysis must be re-oxidized to NAD to maintain glycolysis. But NADH cannot get into the mitochondria for oxidation because it cannot cross the inner mitochondrial membrane. To do so, cytosolic NADH regenerates NAD by reducing oxaloacetate to malate which enters the mitochondrion in exchange for α-ketoglutarate [38]. Malate is re-oxidized to oxalacetate and NADH is oxidized along the electron transport chain to form ATP. Oxaloacetate is transaminated to aspartate which leaves the mitochondrion in exchange for glutamate. The glutamate that enters the mitochondrion is simultaneously transaminated to α-ketoglutarate which leaves the mitochondrion in exchange for malate. In the cytosol, α-ketoglutarate is transaminated to glutamate and aspartate is transaminated to oxaloacetate to complete the cycle. Once formed, glutamate is converted to glutamine and transferred to neurons where it is further processed along a shuttle that is similar to the malate aspartate shuttle to produce the glutamate that is stored in presynaptic vesicles. In GABAergic neurons, glutamate is decarboxylated to GABA and stored [21, 22, 25, 38].

### 4.4 Gamma-aminobutyric Acid

Glutamate and GABA are both taken up by astrocytes following their discharge into the synaptic space. In the astrocytes, about 75% of the glutamate is converted to glutamine and reenters the glutamate/GABA-glutamine cycle while the remaining 25% is oxidatively degraded [38]. GABA generates succinate after it is taken up in the course of two sequential enzymatic reactions, GABA transaminase and succinic semialdehyde dehydrogenase. Succinate, in turn, is converted to α-ketoglutarate along the TCA (Fig. **1**). Thus, astrocytes can use GABA to support glutamine synthesis and sustain neurotransmitter recycling. Inhibition of astrocyte GABA uptake depletes glutamine [21, 22].

## 5 THE LATERAL HABENULA

In contrast to the reduction in glucose utilization in the mPFC, glucose metabolism has been found to be increased in the lateral habenula (LHb), an epithalamic structure, in man and several rodent models of depression [39-42]. The LHb has been described as pathologically hyperactive in major depressive disorder [43]. Activation of the LHb is coupled to the activation of the hypothalamic-pituitary axis (HPA); bilateral lesioning of the LHb abolishes the HPA response to stress. The increased activity in the LHb produced by chronic stress leads to the down-regulation of brainstem dopaminergic and serotonergic neurotransmission and promotes anhedonia and depressive behavior [41, 43, 44]. These neurotransmitter changes also induce the characteristic increase in REM sleep duration found in depression [7, 45]. Pharmacological activation of postsynaptic GABAB receptors opens G protein-activated inwardly rectifying K^+^ (GIRK) channels, which inhibit neuronal activity by generating slow inhibitory postsynaptic currents that significantly suppress LHb neuronal firing rate. Thus, GABAB receptor agonists have been proposed as antidepressant agents [41, 44, 46].

## 6 ANTIOXIDANTS *vs.* STRESS

In rodents, the anhedonia and depressed behavior induced by psychological stress as well as the accompanying signs of oxidative stress and inflammation can be prevented or successfully treated by agents with antioxidative properties. Quercetin, a polyphenol found in many fruits and vegetables, is among the best-studied of these agents [31]. Quercetin significantly suppresses the stress-induced increase of plasma corticosterone and ACTH levels in rats as well as the hypothalamic expression of CRF mRNA [47]. Quercetin also blocks the anxiogenic and depressive-like behaviors induced in mice by CRF [48]. In the CUMS model of depression, quercetin significantly reduces the behavioral signs of stress, and raises superoxide dismutase, catalase and GSH levels while reducing lipid peroxidation and attenuating the expression and levels of the proinflammatory markers IL-6, TNF-α, IL-1β, and COX-2 [31, 49, 50]. Curcumin, another polyphenol, similarly relieves the signs of behavioral depression in rats exposed to CUMS and suppresses microglial activation and overexpression of cytokines IL-1b, IL-6 and TNF-α within the mPFC. It also restores the stress induced reductions in spine density and dendritic retraction within this region [51]. Many other polyphenols, in addition to quercetin and curcumin, have been shown to have therapeutic benefits in rodent models of depression [31]. The critical therapeutic attribute of these agents appears to be their antioxidative power. Apocynin, for example, which is not a polyphenol, alleviates the depressive-like behavior and oxidative stress found in diverse models of depression. Apocynin blocks the epigenetic activation of NADPH oxidase and neutralizes the oxidants that this enzyme generates [52-54]. Yet, neither Apocynin, nor any polyphenol, has advanced to clinical use.

## 7 MAJOR DEPRESSIVE DISORDER

Major Depressive Disorder in man and rodent models of depression share a number of prominent cerebral pathologies: i) reduced prefrontal glucose utilization and glutamate/

GABA-glutamine cycling; ii) the coupled activation of the HPA axis and the LHb; iii) signs of oxidative stress and neuroinflammation; iv) cholinergic hypersensitivity.

- Reduced prefrontal glucose utilization combined with its increase in certain limbic regions has been recognized for some time as a feature of major depression [55, 56]. An inverse relationship has been observed between the reduction in prefrontal metabolism and the severity of depression and it has been proposed that depression may result from the failure of prefrontal inhibition of limbic activity [57, 58]. Depression is judged resistant to treatment when at least two adequate trials with antidepressants from different pharmacologic classes fail to achieve clinical remission [59]. Gathering evidence suggests that reduced prefrontal cortical metabolism predicts treatment refractoriness [58].
- In many depressed patients, HPA activation leads to increased levels of plasma and cerebrospinal fluid cortisol and elevated levels of cerebrospinal fluid CRF [60, 61]. Cortisol normally feeds back on glucocorticoid receptors in the anterior pituitary and hypothalamus to limit the further secretion of CRF and ACTH but these receptors become resistant to feedback regulation in depression possibly in response to the increased levels of pro-inflammatory cytokines found in this disorder such as IL-1, IL-2, IL-6 and TNF-α [61-63]. Pro-inflammatory cytokines may also maintain high levels of plasma cortisol by acting directly on the HPA axis to trigger the release of CRF. CRF may further promote depression by activating the LHb and amplifying cholinergic neurotransmission, another key feature of major depressive disorder [7, 41, 45, 64-67]. On the other hand, persistent stress may exhaust the HPA axis and reduce plasma cortisol levels [68].
- The rapid decay of reactive oxygen species (ROS) makes it challenging to measure the level of oxidative stress in the living brain of patients with major depression [69]. A study using the post-mortem brain found the concentration of the anti-oxidant enzyme Cu/Zn-superoxide dismutase (SOD) increased in homogenates in the frontal region of 7 patients with a history of recurrent depression. A trend in this direction was also found in the HPC. Increased Cu/Zn superoxidase dismutase activity was considered a response to oxidative stress [70]. However, a subsequent study that measured the expression level of the genes for SOD, catalase and glutathione peroxidase, three major oxidative stress-induced cellular response systems, in the post-mortem prefrontal cortex of depressed patients and control subjects found no significant difference between the two groups [71]. More recently, Schiavone *et al.*, analyzed post-mortem frontal cortical tissue derived from a group of subjects who died from suicide by asphyxia, another group who died by non-suicidal asphyxia and a third group who died from other causes and served as controls [72]. They found that the expression of NOX2, a major cellular source of ROS, was significantly higher in the cortex of subjects who died from suicide by asphyxia than in the other two groups. NOX2 was mainly detected in GABAergic neurons. An increase in the expression of 8-hydroxy-2’-deoxyguanosine, a marker of oxidative DNA damage, was also detected in the cortex of these subjects as well as a significant rise in the level of the pro-inflammatory cytokine, interleukin-6.
  Markers of central inflammation have also been identified in the cerebrospinal fluid, in post-mortem brain tissue and by positive emission tomography in patients with major depressive disorder [73]. CSF levels of IL-6 and TNF-α were higher in these patients than in control subjects. CSF levels of IL-6 were increased in subjects who had attempted suicide regardless of their psychiatric diagnosis. Translocator protein, considered a PET marker of monocyte recruitment, was elevated in the anterior cingulate cortex and temporal cortex. The expression of TNF-α mRNA, monocyte chemoattractant protein 1 mRNA and toll-like receptors 3 and 4 was also increased in post-mortem brain. Toll-like receptors 3 and 4 mediate the activation of microglia and increase the production of proinflammatory cytokines.
- A long-standing thesis proposes that depression is mediated by the predominance of central cholinergic over catecholaminergic neurotransmission [45]. Increased cholinergic activity is held to account for the short REM sleep latency and prolonged REM sleep duration observed in major depressive disorder much as it is following psychological stress in rodents [7, 45]. Depressive behavior and REM sleep can be induced in both rodents and men by raising the level of acetylcholine in the synaptic cleft with acetylcholinesterase inhibitors, like physostigmine, or directly with cholinergic muscarinic receptor agonists, like arecoline [45]. In both rodents and men, scopolamine, a nonspecific muscarinic cholinergic antagonist, relieves depression and suppresses REM sleep. Clinical trials demonstrate rapid onset anti-depressant effects with intravenous scopolamine [74, 75]. Similarly, a single, low-dose injection of scopolamine in rodents rapidly induces an antidepressant-like behavioral response in several models of stress [45]. Scopolamine, like ketamine and the psychedelic psilocybin, two other rapidly acting antidepressants, appears to act by triggering the central release of glutamate [76].

## 8 RAPIDLY ACTING ANTIDEPRESSANTS

### 8.1 Scopolamine

Preclinical studies reveal that scopolamine acts as an antagonist at type 1 muscarinic cholinergic receptors located on somatostatin GABAergic interneurons in the mPFC. In mice, the knockdown of these receptors attenuates the anti-depressive effects of scopolamine [77]. The actions of scopolamine on somatostatin interneurons disinhibit pyramidal glutamatergic neurons and, as determined by microdialysis, rapidly increase extracellular glutamate levels in mPFC. This, in turn, stimulates glutamate AMPA receptors and triggers synaptic signaling pathways which activate the mammalian target of rapamycin complex 1 (mTORC1), increase the number and function of dendritic spines and promote synaptogenesis [78]. AMPA receptor antagonists block the anti-depressant effects of scopolamine.

### 8.2 Ketamine

As with scopolamine, clinical trials demonstrate the rapid antidepressant effects of ketamine and the efficacy of ketamine in treatment-resistant depression, bipolar disorder and suicidal ideation [79]. A single subanesthetic dose of ketamine given to treatment-resistant depressed patients can induce a rapid and sustained antidepressant response within hours that can last as long as a week [80].

Preclinical work demonstrates that the antidepressant actions of ketamine are blocked by the chemogenic inhibition of prefrontal cortex interneurons or knockdown of GluN2B NMDAR subunits on somatostatin (SST) and parvalbumin (PV) interneurons but not those on PFC glutamatergic neurons [81, 82]. The inhibition of GABAergic neuronal activity by ketamine suppresses the tonic release of GABA and this, in turn, disinhibits the glutamatergic neurons in the mPFC and increases the release of glutamate. Glutamate acts on AMPA of receptors to increase the release of brain-derived neurotrophic factor (BDNF) binding to its receptor, tropomyosin-related kinase receptor type B (TrkB), and triggering signaling cascades that activate mTORC1 and synaptogenesis [82, 83]. Glutamate and BDNF regulate each other. Glutamate increases the transcription and secretion of BDNF and BDNF increases the release of glutamate. BDNF also regulates the expression of NMDA receptor subunits [84]. Reduced levels of BDNF messenger RNA (mRNA) and protein are found in post-mortem brains of depressed patients [85]. Rodent magnetic resonance spectroscopy (MRS) studies reveal that both ketamine and scopolamine transiently increaseglutamate-glutamine cycling and glutamate neurotransmission in the frontal cortex. This has also been demonstrated in healthy and depressed humans [86]. Ketamine transiently increases cortical glutamate release in a manner that is related to the magnitude of its antidepressant effects [79]. Ketamine increases glucose metabolism, oxygen consumption and blood flow in healthy humans throughout the cortex [79]. Although ketamine and scopolamine stimulate a rapid increase in synaptic glutamate release and cycling, their enduring antidepressant effects are likely dependent on the synaptogenesis induced by the activation of mTORC1 [87, 88].

### 8.3 Electroconvulsive Therapy

Electroconvulsive therapy (ECT) has been used for its rapid actions in treatment-resistant depression and suicidal ideation for over 80 years [89]. The effectiveness of ECT was at one time attributed to enhanced GABA neurotransmission [90]. A GABAergic deficit in depression was implied by the observation that clinically effective antidepressants and shock treatments both enhanced GABAB binding [91]. However, current studies employing MRS fail to identify a difference in brain GABA levels between depressed patients and healthy controls nor a change in these levels following ECT [92, 93]. The in-vivo concentration of GABA in the human brain is less than one-half that of glutamine [94]. In addition, technical problems interfere with the specific measurement of GABA. In standard 1H- MRS of GABA, the signal also includes additional macromolecules and the measurement is denoted as GABA+ [92]. In contrast, many, but not all, MRS studies do identify reduced brain levels of glutamate or glutamate and glutamine combined (Glx) prior to treatment in depressed subjects and a rise in the level of these amino acids following treatment with ECT [89].

## 9 GAMMAHYDROXYBUTYRATE

The nocturnal application of gammahydroxybutyrate (GHB) may correct the major metabolic derangements found in major depressive disorder. GHB has 4 pharmacological features that are pertinent to the treatment of this disorder.

- Firstly, like GABA, GHB is metabolized to succinate and thus it is able to replenish TCA intermediates and maintain glutamate/GABA-glutamine cycling at normal levels [95] (Fig. **1**). In freely moving rats, studies using microdialysis reveal that low doses of GHB infused into the hippocampus increase extracellular levels of glutamate. In-vitro studies demonstrate that glutamate efflux is increased in hippocampal synaptosomes exposed to GHB [96]. In normal human subjects, MRS studies show that a single nocturnal dose of GHB significantly increases the morning glutamate signal in the anterior cingulate cortex together and that this is associated with an improvement in global vigilance [97]. By maintaining normal levels of flux through the TCA, succinate may also restore normal levels of energy (ATP) to the depressed brain.
- Secondly, GHB is a GABAB agonist and, like baclofen, it may block the activation of the LHb and the coupled activation of the HPA axis during a major depressive episode [46, 98]. GHB acts on GABAB receptors to activate GIRK channels and to hyperpolarize and inhibit the largely glutamategic neurons in the LHb much like it hyperpolarizes glutamatergic thalamocortical neurons to produce slow wave sleep [41, 99]. Agonistic actions at GABAB receptors may impede the activation of the LHb axis and the HPA axis by inhibiting the activity of CRF neurons [100]. This may explain the significant attenuation of the normal morning rise in cortisol levels in healthy humans produced by the nocturnal administration of GHB [101]. Given during the day, GHB effectively reduces the hypercortisolism of alcohol withdrawal [102]. By reducing and restoring normal cortisol levels, GHB may attenuate the oxidative stress and neuroinflammation induced by the increased release of cortisol during stress.
- Thirdly, and more to the point, GHB’s metabolism generates NADPH, the cell’s key antioxidant cofactor. GHB has been shown to block the oxidant effects of NADPH oxidase, a generator of the superoxide radical and to suppress oxidative stress [103, 104]. GHB is also a histone-3-acetylase inhibitor and as such inhibits the expression of NADPH oxidase [105, 106].
- Fourthly, accumulating evidence indicates that the cholinergic muscarinic receptor is hyperresponsive in depression and euthymic subjects genetically predisposed to depression [107]. As noted earlier, scopolamine, a cholinergic muscarinic receptor antagonist rapidly relieves depression. GHB, in contrast, acts as a muscarinic cholinergic receptor agonist. It can shorten the REM sleep latency in depressed patients and induce REM sleep in patients in remission whose sleep architecture is normal [108, 109]. However, as studies in narcolepsy reveal, the repeated nightly use of GHB downregulates cholinergic muscarinic receptor sensitivity and restores normal sleep latencies [110]. Cholinergic muscarinic receptor supersensitivity is a characteristic feature of narcolepsy [109]. GHB’s agonistic actions on the GABAB receptor have another pharmacological effect that is relevant to the treatment of the hypercholinergic depressive state [111]. Cholinergic neurons discharge in bursts at maximal rates during active waking and paradoxical sleep. They virtually cease fire during slow-wave sleep. GHB induces slow-wave sleep [109].

Impaired sleep is both a risk factor and a symptom of depression [112]. In a clinical trial of a CRH type 1 receptor antagonist in depressed patients, mood improved in parallel with an increase in slow-wave sleep and a decrease in REM sleep density [113]. GHB, acting on GABAB receptors, may also block the actions of CRF [100]. The use of GHB at night in depressed patients may reestablish the restorative properties of sleep [109].

## CONCLUSION

The rapidly acting antidepressant agents, scopolamine, ketamine and ECT, all act by stimulating an exhausted metabolic system to increase glutamate transmission and glutamate/GABA-glutamine cycling. However, the rapidly acting antidepressants are not prophylactic. Remission and relapse are the natural history of depression which is often a life-long illness [114]. Rapidly acting antidepressants are not used to prevent the next episode. The benefits of the rapidly acting antidepressants are short-lived and they must be applied again and again. The nightly use of GHB may alleviate the multiple metabolic derangements of the stressed brain and terminate the endless cycle of remission and relapse.

## LIST OF ABBREVIATIONS

ACTH: Adrenocorticotropic Hormone
BDNF: Brain-derived Neurotrophic Factor
CRF: Corticotropin-releasing Hormone
CUMS: Chronic Unpredictable Mild Stress
DAMPs: Danger-associated Molecular Patterns
ECT: Electroconvulsive Therapy
GR: Glutathione Reductase
GST: Glutathione S-transferase
HMGB1: High Mobility Group Box 1
HPA: Hypothalamic-pituitary Axis
HPC: Hippocampus
HSP: Heat Shock Proteins
MRS: Magnetic Resonance Spectroscopy
PV: Parvalbumin
ROS: Reactive Oxygen Species
SOD: Superoxide Dismutase
